# Management of oral lesions in toxic epidermal necrolysis with photobiomodulation: Report of three cases

**DOI:** 10.4317/jced.63141

**Published:** 2025-10-01

**Authors:** Weslay Rodrigues da Silva, Ana Paula de Medeiros Silva, Marcella Maria Santos Cabral, Patrícia Batista Lopes do Nascimento, Daniela Siqueira Lopes, Kaline Romeiro

**Affiliations:** 1 Department of Oral and Maxillofacial Pathology, School of Dentistry, Postgraduation Program in Dentistry, Universidade de Pernambuco, Recife, Brazil; 2 Department of Dentistry, Faculty of Dentistry, Postgraduation Program in Dentistry, University of Pernambuco, Recife, Brazil; 3 Division of Dentistry, Dr. Nise da Silveira Women’s Hospital, Maceió, AL, Brazil; 4 Department of Dentistry, Federal University of Alagoas, Maceió, AL, Brazil; 5 Division of Dentistry, Pelópidas Silveira Hospital, Recife, PE, Brazil; 6 Department of Endodontics, Faculty of Dentistry, Facol University Center (UNIFACOL), Vitória de Santo Antão, PE, Brazil

## Abstract

Toxic epidermal necrolysis (TEN) is a mucocutaneous hypersensitivity reaction to certain drugs and/or infections that can lead to multisystem involvement. The oral cavity is affected in almost all cases. The aim of this study was to report three clinical cases of TEN managed with photobiomodulation therapy (PBMT) and antimicrobial photodynamic therapy (aPDT) for the treatment of oral lesions. All patients presented with oral ulcers at various locations. Treatment consisted of oral hygiene with 0.12% chlorhexidine digluconate combined with PBMT applied to strategic sites and aPDT applied to areas of secondary infection. The proposed therapeutic approach resulted in significant clinical improvement of the oral lesions, even prior to improvement of skin manifestations, including pain relief and accelerated tissue healing. These findings suggest that PBMT and aPDT may be effective in improving the oral condition of patients with TEN.

** Key words:**Toxic epidermal necrolysis, Photobiomodulation therapy, Antimicrobial photodynamic therapy.

## Introduction

Toxic epidermal necrolysis (TEN) is a severe mucocutaneous hypersensitivity reaction to certain drugs and/or infections that can result in multisystem involvement [1]. The oral cavity is affected in most cases. However, because of the rarity of the condition, standardized protocols for the management of mucosal lesions, including those involving the oral mucosa, are lacking. A promising therapeutic approach consists of the use of photobiomodulation therapy (PBMT), owing to its analgesic, anti-inflammatory, and tissue repair-stimulating effects. The combination with antimicrobial photodynamic therapy (aPDT), which promotes broad-spectrum antimicrobial activity, may enhance clinical outcomes and accelerate healing [2,3]. Therefore, this study reports the management of oral lesions in three patients with TEN using PBMT and aPDT.

## Case Report

The case reports were conducted in accordance with the ethical principles outlined in the Declaration of Helsinki. Written informed consent was obtained from all participating patients.

Case 1

**Figure 1:**
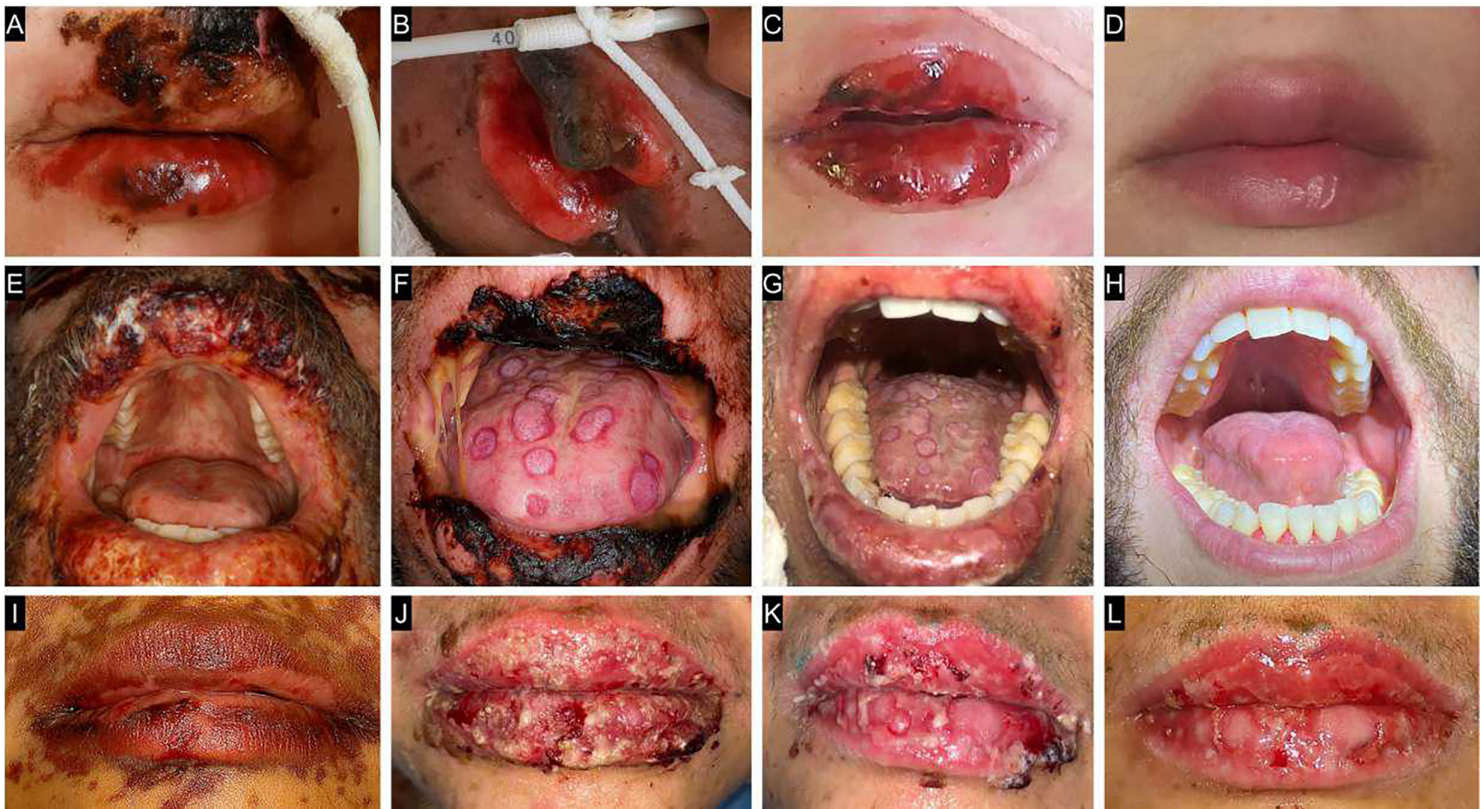
Clinical appearance of patients with toxic epidermal necrolysis. Case 1: A) Initial clinical appearance. B) Clinical appearance after 5 photobiomodulation therapy (PBMT) sessions. C) Clinical appearance after 12 PBMT sessions. D) Final clinical appearance after complete repair. Case 2: E) Initial clinical appearance. F) Clinical appearance 24 hours after the first antimicrobial photodynamic therapy (aPDT) session. G) Clinical appearance after the 5th PBMT session. Note the presence of crusts on the lip and target-shaped lesions on the dorsum of the tongue. H) Final clinical appearance after repair. Case 3: I) Initial clinical appearance. J) Clinical appearance after the 3rd PBMT session. Note the presence of crusts and pustules on the lip. K) Clinical appearance 24 hours after the second aPDT session. L) Final clinical appearance.

A 3-year-old male patient with a history of seizures was started on phenobarbital. Fifteen days after initiating the medication, the patient was admitted to the hospital with fever, cough, odynophagia, conjunctival erythema, and erythematous-violaceous macules. During hospitalization, his clinical condition worsened, and the boy developed flaccid bullae containing serohemorrhagic fluid across the body, along with lesions in the oral cavity. The diagnosis of TEN was established. Extraoral and intraoral examination revealed ulcerated lesions on the vermilion border of the lips covered with crusts, as well as ulcers on the buccal mucosa bilaterally and on the dorsum of the tongue (Fig. 1A,B,C,D).

Case 2

A 31-year-old male patient presented to the hospital’s emergency department with

asthenia, bilateral conjunctivitis, myalgia, cough, odynophagia, and oral pain. The patient had a history of recurrent pharyngitis and had recently taken azithromycin and nimesulide to treat the condition. Shortly thereafter, the patient developed a skin rash, pruritus, and dyspnea. Serological testing was positive for HIV. During hospitalization, the skin lesions worsened, and bullous lesions appeared on the trunk, upper limbs, face, and oral cavity. The diagnosis of TEN was established. Extraoral and intraoral examination revealed ulcerated lesions with crusting on the vermilion border of the lips, as well as ulcers on the buccal mucosa bilaterally, dorsum and lateral borders of the tongue, and hard and soft palate. The tongue lesions had a target-like appearance (Fig. 1E,F,G,H).

Case 3

A 43-year-old female patient presented to the emergency department with fever,

whitish plaques on the tongue and oropharynx, lip edema with bullous lesions, and bilateral conjunctivitis, which had emerged 2 days earlier following the use of clarithromycin for the treatment of pharyngitis. During hospitalization, the patient developed a violaceous rash that initially appeared on the trunk and progressed in a cranio-caudal pattern. There were multiple confluent macules spreading from the abdomen to the lower limbs, some of which became bullous. The oral mucosal lesions also worsened and the diagnosis of TEN was established. Extraoral and intraoral examination revealed ulcerated lesions with crusting on the vermilion border of the lips, as well as ulcers on the buccal mucosa bilaterally, hard and soft palate, and dorsum and lateral border of the tongue. Some of the tongue lesions had a target-like appearance (Fig. 1I,J,K,L).

Management and Monitoring

Due to his age and the severity of his clinical condition, patient 1 was the only one who required enteral feeding via a feeding tube. Patients 2 and 3 were able to maintain oral feeding. Only patient 1 experienced long-term sequelae following recovery, suffering vision loss.

The oral medicine team implemented an oral hygiene protocol consisting of gauze soaked in 0.12% chlorhexidine digluconate, applied every 12 hours. This protocol was followed by low-level red laser PBMT and aPDT, depending on the clinical presentation of each case. Patient 1 underwent 17 PBMT sessions applied to the ulcerated sites. Patient 2 underwent 17 PBMT sessions for the ulcers and two aPDT sessions for the dorsum of the tongue due to delayed healing and suspected secondary infection. Patient 3 underwent 10 PBMT sessions for oral ulcers and three aPDT sessions for the lips due to the presence of pustules.

All patients reported clinical improvement after the second application. Complete healing of the oral lesions was observed in all three patients and occurred prior to the resolution of the skin lesions.

For PBMT, the Therapy XT® device (DMC®, São Carlos, Brazil) was used in continuous mode at red wavelength (λ = 660 nm), with a fixed power output of 100 mW and a spot size of 0.028 cm². The laser was applied for 10 seconds per point, delivering 1 Joule of energy per site.

The same device was used for aPDT. The laser was applied for 50 seconds in contact mode over the lesion, delivering 5 Joules of energy per point. Methylene blue gel (0.01%; Chimiolux®, DMC) was used as the photosensitizing agent, with a pre- irradiation time of 5 minutes. The interval between aPDT sessions was 48 hours.

## Discussion

The majority of patients with TEN present with oral mucosal involvement [4]. Lesions on the oral mucosa can have a typical target shape, while on the lip vermilion, exudative cheilitis is common, often followed by blister and crust formation [5]. Ulcers in the oral cavity can become infected, which increases the production of local inflammatory mediators and delays tissue repair [6]. There are no guidelines for the management of oral lesions in TEN. Thus, for oral hygiene, the present patients received 0.12% chlorhexidine digluconate, a broad-spectrum antimicrobial agent, to reduce the risk of secondary infection and to minimize local inflammation, which would delay repair [7]. In addition, the lesions were managed using PBMT and aPDT to improve outcomes. This approach permitted to combine the analgesic, anti-inflammatory, and repair- stimulating capacity of the red laser [8] with the broad-spectrum microbicidal activity of aPDT [1].

The low-intensity laser used in PBMT relieves pain and inflammation, modulates the immune response, and stimulates repair; it is therefore a promising therapeutic modality for the treatment of various oral adverse events [9]. The effects of PBMT include an increase in metabolism and cell proliferation, enzyme activation, induction of DNA and RNA synthesis, modulation of the action potential of nerve fibers, and stimulation of local microcirculation [10]. These effects justify the use of PBMT for the clinical management of the cases discussed here.

There is limited information on the use of aPDT for the management of oral lesions in patients with TEN, thus representing an innovative therapeutic approach. The mechanism of action of aPDT is based on the absorption of light photons that promote the excitation of electrons in the photosensitizing agent. In the presence of molecular oxygen, this excitation produces reactive oxygen species such as singlet oxygen, which cause damage to cellular structures and the death of microorganisms. This mechanism does not induce microbial resistance [3,10].

The combination of PBMT and aPDT provided satisfactory results in cases of oral lesions due to the combination of antimicrobial activity and modulating, reparative, and analgesic properties, promoting more effective management of TEN [10]. The treatment proposed appears to be effective, especially given the severity of cases and early repair compared to extraoral sites. Only the therapies mentioned and oral hygiene were used for the management of oral cavity lesions. In addition to healing, these therapies contributed significantly to improving quality of life, especially of patients 2 and 3, by promoting pain relief and maintaining oral food intake. The latter favors an adequate nutritional status and can accelerate clinical recovery.

## Conclusions

The satisfactory management of oral lesions in patients with TEN demonstrates the effectiveness of PBMT and aPDT, with these therapies accelerating tissue repair, promoting analgesia, and facilitating decontamination. This fact enabled the resolution of oral lesions before the skin lesions and the maintenance of an oral diet, ultimately contributing to the improvement of the patient’s general health status.

## Data Availability

The data that support the findings of this study are available from the corresponding author upon reasonable request.
